# TDP-43 and NEAT long non-coding RNA: Roles in neurodegenerative disease

**DOI:** 10.3389/fncel.2022.954912

**Published:** 2022-10-26

**Authors:** Durairaj Sekar, Deusdedit Tusubira, Kehinde Ross

**Affiliations:** ^1^Centre for Cellular and Molecular Research, Saveetha Dental College and Hospitals, Saveetha Institute of Medical and Technical Sciences (SIMATS), Saveetha University, Chennai, India; ^2^Department of Biochemistry, Mbarara University of Science and Technology, Mbarara, Uganda; ^3^School of Pharmacy and Biomolecular Sciences, Liverpool John Moores University, Liverpool, United Kingdom; ^4^Institute for Health Research, Liverpool John Moores University, Liverpool, United Kingdom

**Keywords:** TDP-43, long non-coding RNA, NEAT1, neurons, paraspeckles, TAR DNA-binding protein 43, nucleic acid therapies, swimming microrobots

## Abstract

Understanding and ameliorating neurodegenerative diseases represents a key challenge for supporting the health span of the aging population. Diverse protein aggregates have been implicated in such neurodegenerative disorders, including amyloid-β, α-synuclein, tau, fused in sarcoma (FUS), and transactivation response element (TAR) DNA-binding protein 43 (TDP-43). Recent years have seen significant growth in our mechanistic knowledge of relationships between these proteins and some of the membrane-less nuclear structures that fulfill key roles in the cell function. These include the nucleolus, nuclear speckles, and paraspeckles. The ability of macromolecular protein:RNA complexes to partition these nuclear condensates through biophysical processes that involve liquid–liquid phase separation (LLPS) has also gained attention recently. The paraspeckle, which is scaffolded by the architectural long-non-coding RNA nuclear enriched abundant transcript 1 (NEAT1) plays central roles in RNA processing and metabolism and has been linked dynamically to TDP-43. In this mini-review, we outline essential early and recent insights in relation to TDP-43 proteinopathies. We then appraise the relationships between TDP-43 and NEAT1 in the context of neuronal paraspeckles and neuronal stress. We highlight key areas for investigation based on recent advances in our understanding of how TDP-43 affects neuronal function, especially in relation to messenger ribonucleic acid (mRNA) splicing. Finally, we offer perspectives that should be considered for translational pipelines in order to improve health outcomes for the management of neurodegenerative diseases.

## Introduction

Neurodegenerative diseases are a diverse set of conditions characterized by the progressive degradation of the structure and function of the central or peripheral nervous systems (Dugger and Dickson, 2017). Major neurodegenerative disorders include Alzheimer’s disease (AD), amyloid lateral sclerosis (ALS; also known as Lou Gehrig’s disease), frontotemporal lobar degeneration (FTLD), Huntington’s disease (HD), and Parkinson’s disease (PD). Collectively, such neurodegenerative disorders are projected to become the second leading cause of death after cardiovascular disease by 2040 (Gammon, 2014). In contrast to rapid neuronal loss caused by metabolic or toxic illnesses, neurodegenerative disorders are characterized by the gradual loss of selectively vulnerable populations of neurons, thus diminishing the functional properties of the brain and peripheral organs (Dugger and Dickson, 2017). Cell death and shrinkage in a specific region of the brain represent a major pathogenic trait in several neurodegenerative illnesses (Dugger and Dickson, 2017).

Pathological deposition of intracellular or extracellular protein aggregates is associated with several neurodegenerative disorders. Depending on the specific protein, these conditions can be grouped into amyloidosis (amyloid-β), α-synucleinopathies (α-synuclein), tauopathies (tau), FUSopathies [Fused in sarcoma (FUS)], and TDP-43 proteinopathies [transactivation response element (TAR) DNA-binding protein 43 (TDP-43)] (Kovacs, 2017). Such protein aggregates, and/or their oligomeric species frequently stress neurons, resulting in neuronal death (Chi et al., 2018).

The TDP-43 proteinopathies include ALS, FTLD-TDP, primary lateral sclerosis, and progressive muscular atrophy and are defined by TDP-43-related pathology (Dugger and Dickson, 2017). The hallmarks of TDP-43 proteinopathies include cytoplasmic accumulation, phosphorylation, ubiquitination, and truncation of TDP-43 into neurotoxic *C*-terminal fragments (Chhangani et al., 2021). Most cases of ALS are sporadic, though 10% of ALS cases are considered to be familial (Taylor et al., 2016). In addition to TDP-43, a further 15 genes harboring mutations have been unequivocally associated with ALS by strong genetic and functional data, as reviewed by Taylor et al. (2016). These include Cu-Zn superoxide dismutase 1 (*SOD1*), FUS, optineurin (*OPTN*), TANK binding kinase 1 (*TBK1*), and *C9ORF72* (Taylor et al., 2016). More recently, 12 additional genomic loci have been implicated in ALS by genome-wide association studies (GWAS), including *KIF5A*, *GPX3*, *TNIP1*, and *UNC13A* (van Rheenen et al., 2016, 2021; Benyamin et al., 2017; Nicolas et al., 2018). A machine learning approach that integrated epigenetic features of motor neurons with ALS GWAS data also revealed a role for *KANK1* in ALS pathology, from 690 ALS-associated genes that were identified (Zhang et al., 2022).

Transactivation response element (TAR) DNA-binding protein 43 is a highly conserved heterogeneous ribonucleoprotein (hnRNP) multi-domain protein first identified as a 43-kDa protein that bound the TAR in human immunodeficiency virus (Ou et al., 1995). Under normal physiological conditions, TDP-43 is subjected to nucleocytoplasmic shuttling while residing predominantly in the nucleus (Ayala et al., 2008). This localization to both nuclear and cytosolic compartments is reflected in the processes regulated by TDP-43, which span messenger ribonucleic acid (mRNA) transcription splicing, maturation, and mRNA transport as well as the formation of stress granules and the regulation of miRNA processing, as reviewed recently by Prasad et al. (2019). Unsurprisingly, therefore, mutations that increase TDP-43 aggregation, increase TDP-43 half-life, or alter TDP-43 interactions with other proteins are thought to contribute to disease pathology in TDP-43 proteinopathies, and over 52 TDP-43 mutations have been linked to disease (Buratti, 2015).

There is growing recognition that TDP-43 undergoes liquid–liquid phase separation, (LLPS) a biophysical process that gives rise to biomolecular condensates reminiscent of an oil-in-vinegar mixture (Conicella et al., 2020; Ditlev, 2021; Schmit et al., 2021; Cascella et al., 2022). Driven by multivalent intermolecular interactions involving proteins and nucleic acids, biophysical LLPS is thought to support thermodynamically favorable formation of membraneless organelles endowed with specific functions within the nucleus and cytoplasm (Banani et al., 2017; Peng et al., 2021). Several such membraneless compartments have been defined in the nucleus, include the nucleolus, nuclear speckle, Cajal body, histone locus body, and paraspeckles which are broadly related by various aspects of RNA processing (Mao et al., 2011), as well as Polycomb bodies, promyelocytic leukemia (PML) bodies and nuclear stress bodies (Courchaine et al., 2016; Sawyer et al., 2019; Corpet et al., 2020; Wang et al., 2021). In this review, we appraise some of these nuclear bodies briefly then comment on recent developments in our understanding of TDP-43 in neurodegenerative disorders, highlighting areas for further research and therapeutic strategies that may help ameliorate disease.

## Nuclear bodies and liquid–liquid phase separation

The nucleolus was established as the site for ribosome biogenesis in the 1960s and through a variety of technological innovations, we now recognize that key nucleolar proteins involved in rRNA transcription shuttle rapidly between the nucleolus and nucleoplasm (Chen and Huang, 2001). Further, the nucleolus consists of three phase-separated subcompartments: multiple copies of the fibrillar center (FC) and the dense fibrillar component (DFC) embedded within the granular component (GC) (Boisvert et al., 2007; Lafontaine et al., 2021). Recent studies indicate the assembly of the DFC is driven by phase-separation mediated by nascent pre-rRNA sorting (Yao et al., 2019), while binding of the abundant nucleolar protein nucleophosmin (NPM1) to proteins and RNA drives the formation of phase separated multicomponent liquid-like droplets in the GC matrix (Mitrea et al., 2016). Importantly, the GC also functions as a quality control center into which misfolded proteins are sequestered reversibly during periods of cell stress in order to prevent their aggregation, allowing subsequent heat shock protein 70–dependent refolding during recovery from the stress (Frottin et al., 2019). Interestingly, nucleolin, another abundant nucleolar protein, was recently shown to alleviate TDP-43-related toxicity, at least in yeast and HEK293 cell models, apparently by facilitating the nuclear retention of TDP-43, thus preventing its accumulation as toxic cytoplasmic condensates (Peggion et al., 2021).

Nuclear speckles (interchromatin granule clusters) are highly dynamic structures enriched for transcription factors, chromatin remodeling factors and other proteins that enable nuclear speckles to function as a hub for coordinated regulation of the various steps associated with gene expression (Galganski et al., 2017). These include gene positioning, chromosome localization, chromatin modification, transcription, splicing, 3′ end processing, and mRNA modification (Galganski et al., 2017; Faber et al., 2022). Notably, in an attempt to clarify exactly what should be called a nuclear speckle and based on recent findings, Ilik and Aktas, 2021 proposed to define as nuclear speckles by LLPS and the presence of two proteins SON and serine/arginine repetitive matrix protein 2 (SRRM2), that are rich in intrinsically disordered regions.

Cajal bodies (CB) are multifunctional biomolecular condensates that support the maturation of small nuclear ribonucleoproteins (snRNPs). The snRNPs are multimeric protein-RNA complexes that form the spliceosome and it is thought that snRNPs are concentrated in CB in order to promote the assembly of the individual monomers into higher order complexes (Klingauf et al., 2006; Morris, 2008; Shaw et al., 2008). Cajal bodies are defined by the presence of coilin, although other proteins such as survival motor neuron protein (SMN; the spinal muscular atrophy disease gene product), Geminis, Nopp140, and fibrillarin, as reviewed in Lafarga et al. (2017). Loss of coilin is semi-lethal, with about half of coilin knockout mice dying late in gestation (Walker et al., 2009). The reproductive fecundity of the mice was reduced, with smaller litter sizes and fewer litters overall (Walker et al., 2009). Thus, it appears that CB function has evolved to be somewhat robust such that in the absence of coilin, the “residual” CB can maintain levels of function that support viability. Interestingly, the localization of small Cajal body RNAs (scaRNAs) was recently shown to be regulated by TDP-43 and this in turn regulated the site-specific 2′-*O*-methylation of U1 and U2 small nuclear RNAs (Izumikawa et al., 2019).

Interestingly, early work showed CB interactions with replication-dependent histone (RDH) loci (Shopland et al., 2001). These histone locus bodies (HLBs) have now been linked to the formation of a ternary complex with CB and RNA polymerase II, facilitating 3′-end processing of RDH genes (Imada et al., 2021; Suzuki et al., 2022). The dependence of these HLBs on histone pre-mRNA transcripts has led to the histone pre-mRNA transcripts being classified as architectural or scaffolding RNAs (Shevtsov and Dundr, 2011; Chujo and Hirose, 2017).

Three long non-coding RNAs (lncRNAs) have been also been categorized as scaffolding or architectural RNAs associated with nuclear bodies in human cells (Chujo and Hirose, 2017). These are (a) mammalian nuclear enriched abundant transcript 1 isoform 2 (NEAT1_2) lncRNA in the paraspeckle, (b) ribosomal intergenic spacer (IGS) lncRNA that drives amyloid body assembly for local nuclear protein synthesis (Theodoridis et al., 2021), and (c) human satellite III (SatIII) lncRNA in the nuclear stress body (Valgardsdottir et al., 2005, 2008). In addition, *Drosophila* heat shock RNA (Hsr) omega in the omega speckle (Mallik and Lakhotia, 2009) and the fission yeast lncRNA meiRNA in the Mei2 dot (Yamashita et al., 1998) also meet the criteria to be considered architectural RNAs (Chujo and Hirose, 2017).

In healthy cells, TDP-43 resides in the nucleus and regulates RNA processing and translation, functions that are lost following of mis-localization of TDP-43 as cytoplasmic inclusions (Bjork et al., 2022). Both the loss of function associated with nuclear depletion and the gain of toxicity due to cytoplasmic accumulation are implicated in the pathogenic effects of TDP-43 (Chhangani et al., 2021). Recent studies have started to uncover complex relationships between TDP-43 and the lncRNA NEAT1 in TDP-43 proteinopathies, and we now turn to this theme.

## Transactivation response element DNA-binding protein 43 proteinopathies: A brief overview

Landmark proteomics studies on post-mortem brain extracts from FTLD and ALS patients in 2006 uncovered TDP-43 as a key protein associated with FTLD, specifically ubiquitin-positive but tau- and alpha-synuclein negative FTLD (Arai et al., 2006; Neumann et al., 2006). This predominantly nuclear protein that was known to bind the TAR element of the human immunodeficiency virus has since been implicated in the pathogenesis of multiple neurodegenerative diseases collectively known as TDP-43 proteinopathies (de Boer et al., 2020; Huang et al., 2020; Carlos and Josephs, 2022). Early studies observed TDP-43 cytoplasmic inclusions in ALS and AD (Arai et al., 2006; Igaz et al., 2008). Further, a transgenic mouse model with moderate human TDP-43 overexpression showed intranuclear and cytoplasmic phosphorylated TDP-43 aggregates in neurons. Importantly, these mice showed axonal and myelin degeneration which were reflected in gait abnormalities and early mortality (Xu et al., 2010). Some TDP-43 aggregates localize to mitochondria and, crucially, inhibition of TDP-43 mitochondrial localization suppresses TDP-43 neuronal toxicity (Mori et al., 2008; Wang et al., 2016). Recent studies have now revealed the structural basis for these observations in ALS as aggregated TDP-43 sequesters selected microRNAs (miRNAs) and proteins, leading to altered expression of nuclear-genome-encoded mitochondrial proteins (Zuo et al., 2021). This in turn generates a global mitochondrial imbalance that drives oxidative stress (Zuo et al., 2021). Interestingly, TDP-43 were shown to form amyloid-like filaments in the brains of patients who had ALS with FTLD (Arseni et al., 2022) so it will be intriguing to determine how the filaments drive protein and miRNA sequestration. In addition, very recent work indicated that brain samples from TDP-43 proteinopathies as well as tauopathies and synucleinopathies are characterized by amyloid fibrils consisting of a 135-amino acid C-terminal fragment of transmembrane protein 106B (TMEM106B) (Chang et al., 2022; Jiang et al., 2022; Schweighauser et al., 2022). However, the relationships of these TMEM106B fibrils to disease pathology remains to be established.

In an early study of protein:RNA interactions in the post-mortem brain tissue by Tollervey et al. (2011), most TDP-43 binding was found on introns, long ncRNAs and intergenic RNAs. Similar results were observed in cultured cells. In addition, the authors found that TDP-43 regulates pre-mRNA splicing, generating alternative mRNA isoforms for several proteins involved in neuronal development or disease (Tollervey et al., 2011). What has become clear more recently is that TDP-43 suppresses the incorporation of poorly conserved cryptic exons into mRNA, and TDP-43 deficiency in the nucleus is associated with the generation of mis-spliced transcripts that are targeted for nonsense-mediated decay (Ling et al., 2015; Humphrey et al., 2017; Jeong et al., 2017; Sun et al., 2017).

## Transactivation response element DNA-binding protein 43 and Alzheimer’s disease

In addition to ALS and FTLD, roles for TDP-43 in the pathology of AD have also been established. Several studies have observed TDP-43 immunoreactivity in a subset of AD cases with neuroanatomical distribution pattens that were broadly distinct from those observed in FTLD (Amador-Ortiz et al., 2007; Higashi et al., 2007; Uryu et al., 2008; Arai et al., 2009; Kadokura et al., 2009; King et al., 2010; Davidson et al., 2011; McAleese et al., 2017). The findings are summarized in Table 1. Subsequent studies have linked TDP-43 with lower cognitive function in AD patients in which hippocampal sclerosis has been reported (Nag et al., 2015). Importantly, even when subjects had comparable levels of AD pathology based on Braak staging, TDP-43 positive cohorts were much more likely to have cognitive malfunction compared to their TDP-negative counterparts, and the effects of TDP-43 appeared to be independent of hippocampal sclerosis (Josephs et al., 2014). Of note, TDP-43 deposition appears to be part of the normal physiological response during aging with neuronal cytoplasmic inclusions observed in 36% in the brains of cognitively normal subjects aged 71–100 years (Arnold et al., 2013).

**TABLE 1:** Transactivation response element (TAR) DNA-binding protein 43 (TDP-43) positive immunoreactivity in Alzheimer’s disease (AD) patients.

TDP-43 positive patients/Total number of patients	Percentage	References	Comments
9/85	11	Davidson et al., 2011	Early onset AD
19/93	20	Amador-Ortiz et al., 2007	
47/182	26	Uryu et al., 2008	
24/84	29	Davidson et al., 2011	Late onset AD
5/16	31	Kadokura et al., 2009	
5/15	33	Higashi et al., 2007	
19/53	36	Arai et al., 2009	
14/25	56	Arai et al., 2009	
34/46	74	McAleese et al., 2017	
6/8	75	King et al., 2010	

Early work revealed that the amygdala was particularly vulnerable to TDP-43 in advanced AD region (Hu et al., 2008). Further, detailed analysis of TDP-43 distribution across 14 brain regions led to the six-stage scheme in which TDP-43 immunoreactivity is limited to the amygdala at stage 1 and extends progressively to the entorhinal cortex and subiculum (stage 2), the dentate gyrus of the hippocampus and occipitotemporal cortex (stage 3), insular cortex, ventral striatum, basal forebrain, and inferior temporal cortex (stage 4), substantia nigra and midbrain tectum (stage 5), and basal ganglia and middle frontal cortex (stage 6) (Josephs et al., 2016). Interestingly, the distinct distribution patterns of TDP-43 have been observed in relation to these stages: stages 4–6 were linked to “typical” TDP-43 immunoreactive inclusions defined by neuronal cytoplasmic inclusions, neuronal intranuclear inclusions, and dystrophic neurites, while stages 1–3 associated with a “type-β” deposition in which TDP-43 immunoreactivity occurs adjacent to tau-positive neurofibrillary tangles in the same neuron (Josephs et al., 2019). However, both types were associated with smaller amygdala and hippocampal volumes and such volumetric differences may be predictive of TDP-43 status while patients are alive. For further details on the relationships between TDP-43 and classical neuropathological features of AD such as amyloid-β and tau oligomers, the reader is referred to an excellent recent review by Meneses et al. (2021) that also includes a helpful depiction of the TDP-43 distribution pattern in AD.

## Transactivation response element DNA-binding protein 43 clearance and altered splicing

One mechanism of disease recently implicated in ALS and FTLD-TDP links the loss of nuclear TDP-43 to mis-splicing and depletion of stathmin 2 (*STMN2*), a regulator of the microtubule cytoskeleton (Klim et al., 2019; Melamed et al., 2019; Prudencio et al., 2020; Pickles et al., 2022). Given the importance of microtubules for normal neuron function, it is noteworthy that two independent studies have now demonstrated that stathmin deficiency leads to motor dysfunction in mouse models (Guerra San Juan et al., 2022; Krus et al., 2022).

**FIGURE 1:**
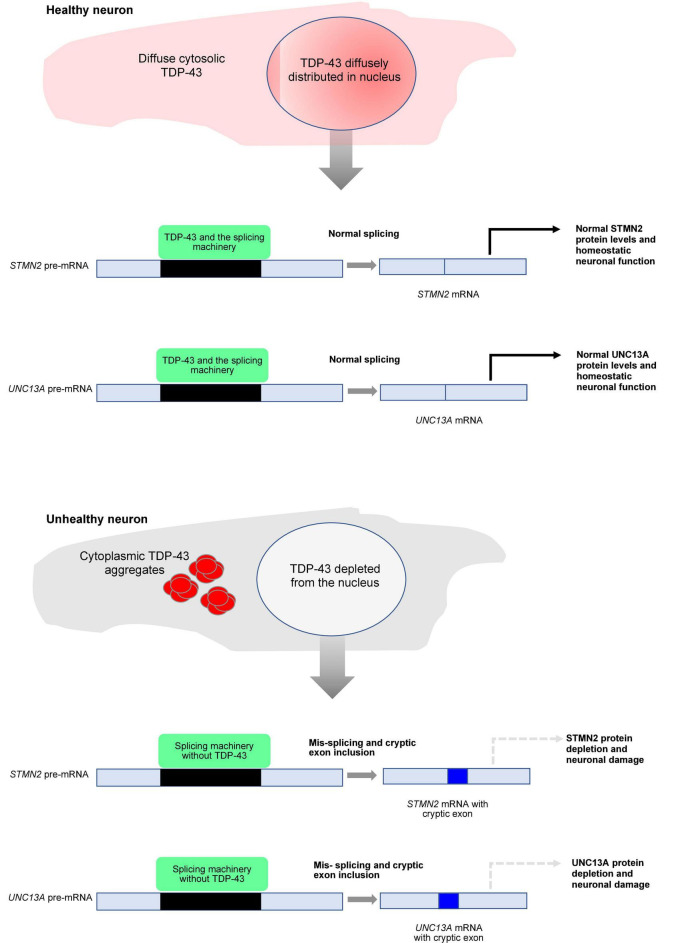
Schematic depiction of transactivation response element (TAR) DNA-binding protein 43 (TDP-43) mis-localization and neurodegeneration. In healthy neurons, TDP-43 is localized predominantly to the nucleus but is also maintained in the cytoplasm at comparatively low levels *via* nucleocytoplasmic shuttling. Nuclear TDP-43 facilitates appropriate splicing of target transcripts, leading to normal levels of protein expression (*solid arrows*; for simplicity only *STMN2* and *UNC13A* are shown). Upon aggregation of TDP-43 in the cytoplasm, TDP-43 functions in the nucleus are lost, resulting in the incorporation of cryptic exons during target transcript splicing. The resulting decrease in target protein expression (*dashed arrows*) in turn drives neuronal malfunction and degeneration associated with disease.

Loss of nuclear TDP-43 has also been linked to mis-splicing events that decrease the expression of UNC13A in neurons (Brown et al., 2022; Ma et al., 2022). The UNC13A protein is critical for normal synaptic function and altered *UNC13A* splicing introduces a cryptic exon that leads to premature stop codons (Brown et al., 2022; Ma et al., 2022). The relationships between TDP-43 and cryptic exon splicing leading to neuronal dysfunction are summarized in Figure 1. Importantly, single-nucleotide polymorphisms (SNPs) associated with FTLD and ALS risk were found in the region containing the cryptic exon (Brown et al., 2022; Ma et al., 2022). Genetic variants associated with disease risk correlated with elevated levels of *UNC13A* cryptic exon inclusion. Further, the SNPs influenced TDP-43 binding to *UNC13A* pre-mRNA and enhanced cryptic exon inclusion when *UNC13A* minigenes were transcribed (Brown et al., 2022; Ma et al., 2022). The conclusions reached in these studies was not so much that the *UNC13A* risk variants play a causative role in disease but rather, against a backdrop of TDP-43 clearance from the nucleus, insufficient TDP-43 binding to the cryptic exon permits incorporation of cryptic exon into pre-mRNA.

The study by Tollervey et al. (2011) also detected substantially more TDP-43 binding to lncRNA NEAT1 in FTLD-TDP samples, possibly due to increased NEAT1 expression. Given that NEAT1 serves as an architectural lncRNA for paraspeckle formation, we now turn to the relationships between NEAT1, TDP-43, and paraspeckles in the neuronal nucleus.

## Nuclear enriched abundant transcript 1 and transactivation response element DNA-binding protein 43 proteinopathy in the neuronal paraspeckle

Early work by Hutchinson et al. (2007) led to the identification of NEAT1 and NEAT2 (also known as MALAT-1). Subsequent independent work from Hirose and Spector groups defined two NEAT1 isoforms transcribed from the multiple endocrine neoplasia I (MEN I) genomic locus, a short isoform of 3.7 kb (initially MENε, now known as NEAT1_1) and a much longer transcript of 23 kb (initially MEN β, now NEAT1_2) (Sasaki et al., 2009; Sunwoo et al., 2009). These studies also provided initial evidence linking NEAT1 lncRNAs to paraspeckles (Sasaki et al., 2009; Sunwoo et al., 2009). Further work confirmed the involvement of NEAT1 in paraspeckle assembly, with NEAT1_2 in particular serving as a scaffold or architectural lncRNA to support paraspeckle formation (Clemson et al., 2009; Souquere et al., 2010).

In neurons, upregulation of NEAT1 was linked to neuronal and oligodendrocyte maturation (Mercer et al., 2010) but it was work by Tollervey et al. (2011) that established a possible connection to FTLD by showing that NEAT1 and MALAT-1 bound TDP-43 in post-mortem human brain samples. Further evidence for NEAT1 involvement in neurodegenerative disorders came from studies by Nishimoto et al. (2013) whose analysis of 633 human spinal motor neurons derived from six ALS patients revealed elevated expression of NEAT1_2 in the early stages of the disease. The investigators also confirmed the ability of TDP-43 to bind NEAT1_2 and colocalize with NEAT1_2 in paraspeckles (Nishimoto et al., 2013). These findings together gave rise to the notion of NEAT1_2 lncRNA as a scaffold for the assembly of macromolecular protein and RNA complexes in the nuclei of ALS motor neurons in the early phase of the disease. Importantly, elevated expression of NEAT1 has also been observed in other neurodegenerative diseases, including frontotemporal dementia, AD, HD, and PD, as reviewed in An et al. (2018).

These early studies thus revealed a clear axis of neurodysregulation associated with NEAT1_2 and TDP-43. What was less clear was whether NEAT1_2 roles were pathogenic or a compensatory effect to stave off neuronal death. Two main concepts have now begun to emerge, however. First, that LLPS appears to be the driving force for paraspeckle assembly, with the middle domain in particular recruiting the essential paraspeckle proteins (PSPs) non-POU domain-containing octamer-binding protein (NONO) and SFPQ (splicing factor proline and glutamine rich), with NONO forming dimers that support oligomerization with other PSPs (Yamazaki et al., 2018). From a biophysical perspective, the spatial arrangement of NEAT1 and the PSPs appears to mimic amphipathic block copolymer micelles in which connected hydrophilic and hydrophobic polymers self−assemble into spherical and cylindrical nanostructures in water (Yamazaki et al., 2021). Secondly, that upregulation of NEAT1 and of paraspeckles exerts a protective effect on neurons, to which we now turn.

## Nuclear enriched abundant transcript 1: Transactivation response element DNA-binding protein 43 as protective partners in the neuronal stress response

Early work from Nakagawa et al. (2011) found widespread constitutive expression of NEAT1_1, whereas NEAT1_2 expression was limited to specific cells in the stomach and intestine. There was no obvious phenotype to *NEAT1-*deficient mice, beyond the loss of paraspeckles although subsequent work revealed a high expression of NEAT1 in the corpus luteum of mouse ovaries, impaired formation of luteal tissue, low serum progesterone, and a stochastic inability to become pregnant (Nakagawa et al., 2014). In contrast, no essential physiological roles were identified from studies on mice specifically deficient in NEAT1_1 (Adriaens et al., 2019). It would therefore be interesting to explore the effects of NEAT1_1 depletion in other mammalian orders, especially in primates or primate tissues, to uncover potential functions in these organisms.

**FIGURE 2:**
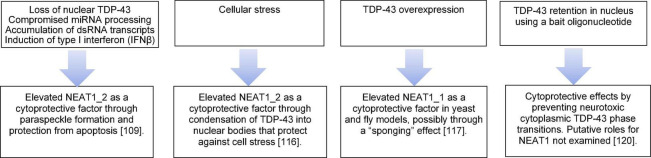
Cytoprotection associated with transactivation response element (TAR) DNA-binding protein 43 (TDP-43) and nuclear enriched abundant transcript 1 (NEAT1) function. Schematic representation of relationships linking TPD-43 and NEAT1 to protection from toxicity in TDP-43 proteinopathy.

Notably, as illustrated in Figure 2 and discussed below, NEAT1 nonetheless appears to exert cytoprotective effects in diverse models. Neurons do not express NEAT1_2 under basal conditions and are devoid of paraspeckles at least *in vitro* (Shelkovnikova et al., 2018). Unsurprisingly, therefore, mice lacking NEAT1 show no evidence of neuronal loss, neuroinflammation, or gross synaptic dysfunction (Kukharsky et al., 2020). On the other hand, Shelkovnikova et al. (2018) recently observed paraspeckles in a small cohort of ALS patients (two with TDP-43 pathology, four with C9ORF72 pathology, and seven with sporadic ALS), with ∼30% of spinal cord neurons displaying paraspeckles compared to no paraspeckles in neurons from healthy control patients. An associated increased NEAT1 expression was also reported (Shelkovnikova et al., 2018). Given that key features of TDP-43 proteinopathy include clearance of the protein from the nucleus with concomitant accumulation and aggregation in the cytoplasm (Arai et al., 2006; Neumann et al., 2006), it was notable that silencing of TDP-43 in SH-SY5Y neuroblastoma cells raised the number of paraspeckles per nucleus and expression of the paraspeckle-specific NEAT1 isoform, NEAT1_2 (Shelkovnikova et al., 2018). More importantly, because TDP-43 can enhance miRNA biogenesis (Buratti et al., 2010; Kawahara and Mieda-Sato, 2012), the formation of paraspeckles in TDP-43-deficient neuroblastoma cells appears to serve a protective role by marshaling the pri-miRNA processing machinery to sustain the generation of miRNAs that target pro-apoptotic genes (Bottini et al., 2017; Jiang et al., 2017; Shelkovnikova et al., 2018). However, although some TDP-43-binding mRNAs were altered in the serum and cerebrospinal fluid (CSF) of sporadic ALS patients (Freischmidt et al., 2013), the relationships between TDP-43 and motor neuron miRNA expression has not been fully established.

The idea that the upregulation of NEAT1_2 and paraspeckle abundance exerts a neuroprotective role is further supported by recent work from Wang et al. (2020). Distinct triggers of cell stress, including arsenite and leptomycin B (an inhibitor of nuclear export), induced TDP-43 to form dynamic, reversible liquid-droplet-like nuclear bodies in mammalian cells, including mouse neurons (Wang et al., 2020). Importantly, studies on fly models of ALS-related function suggested that the TDP-43 nuclear bodies were neuroprotective *in vivo*. Stress also raised the levels of NEAT1 RNA in neurons, which in turn supported the condensation of TDP-43 through mechanisms that exploited LLPS. One particular ALS-associated TDP-43 mutation D169G, but not other mutations examined, reduced NEAT1 mediated TDP-43 LLPS. Importantly, the TDP-43 nuclear bodies appear to exert a cytoprotective effect in human embryonic kidney (HEK293T) cells as well as *Drosophila* neurons (Wang et al., 2020). Clearly, it will be crucial to determine whether NEAT1-induced TDP-43 nuclear bodies formed in response to neuronal stress suppresses the incorporation of cryptic, neurodegenerative *STMN2* and *UNC13A* exons (Klim et al., 2019; Melamed et al., 2019; Prudencio et al., 2020; Brown et al., 2022; Ma et al., 2022).

Depletion of TDP-43 drives paraspeckle assembly through elevation of NEAT1_2 expression that is mediated by type 1 interferon (Shelkovnikova et al., 2018). This upregulation of NEAT1_2 expression appears to result from the accumulation of miRNA species (Shelkovnikova et al., 2018). On the other hand, more recent work has suggested that overexpression of full length TDP-43 that localizes to the nucleus (but not TDP-43 lacking the nuclear localization signal) can elevate NEAT1_1 expression in SH-SY5Y neuroblastoma cells and in the central nervous systems (CNS) cortex of a mouse model (Matsukawa et al., 2021). Even though paraspeckles are not observed in cells that express only NEAT1_1, which instead distributes in a diffuse “microspeckle” pattern that does not co-localize with paraspeckle markers in the nucleoplasm (Nakagawa et al., 2011; Li R. et al., 2017), NEAT1_1 alleviated TDP-43-dependent toxicity in yeast and *Drosophila* models of TDP-43 proteinopathy (Matsukawa et al., 2021). One idea proposed for the ability of NEAT1_1 to ameliorate TDP-43 toxicity is that NEAT1_1 binding to TDP-43 may block unwanted interactions of TDP-43 with other nuclear RNAs (Matsukawa et al., 2021). Hence, it will be interesting to determine whether NEAT1_1 can also prevent TDP-43-dependent toxicity in non-human primate models (Uchida et al., 2012).

Work from Mann et al. (2019) recently indicated that a 34-nt TDP-43-binding oligonucleotide abrogated the phase transitions and neurotoxicity of pathologically-relevant TDP-43 inclusions, at least as monitored using optogenetic (i.e., light-dependent) oligomerization strategies. Strikingly, the oligonucleotide apparently helped maintain optogenetic TDP-43 in the nucleus and prevent localization to the cytoplasm, though the mechanisms of this potentially therapeutic axis requires further investigation. Further work is also required to establish whether the action of such oligonucleotides is associated with the induction of paraspeckle formation in neurons. Beyond these studies, there is clearly an urgent need to establish whether TDP-43-binding oligonucleotides can restore the ability of TDP-43 to suppress splicing of the cryptic *STMN2* and *UNC13A* exons implicated in neurodegeneration (Klim et al., 2019; Melamed et al., 2019; Prudencio et al., 2020; Brown et al., 2022; Ma et al., 2022).

The above considerations together suggest complex effects of TDP-43 in supporting neuroprotective responses *via* elevation of NEAT1_2 and NEAT1_2. Further studies are required to validate the therapeutic potential of paraspeckle induction and NEAT1_1 elevation in TDP-43 neurodegenerative disorders. These will need to span zebrafish, mouse and primate models of TDP-43 proteinopathy as have been used in recent studies (Lissouba et al., 2018; Yin et al., 2019; Huang et al., 2021). One important question to address will be whether NEAT1_2 elevation (or more generally, enhancement of paraspeckle numbers) in parallel with increased expression of NEAT1_1 has an additive or synergistic effect on neuroprotection from TDP-43 toxicity.

## Therapeutic horizons: Oligonucleotide approaches

Interestingly, *de novo* paraspeckle assembly appears to be a key feature of ALS TDP-43 proteinopathy (Shelkovnikova et al., 2018). Exposure of SH-SY5Y neuroblastoma cells to enoxacin, a small molecule that promotes miRNA biogenesis, boosted NEAT1_2 and paraspeckle abundance, adding a lncRNA:paraspeckle axis to the mode of action of enoxacin (Shan et al., 2008; Melo et al., 2011; Emde et al., 2015; Shelkovnikova et al., 2018). These paraspeckles appear to serve a protective role in neuroblastoma cells. However, neither enoxacin nor other agents (such as histone deacetylase inhibitors) that induced NEAT1_2 and *de novo* paraspeckles in neuroblastoma cells were able to evoke paraspeckle assembly in human embryonic cell-derived motor neurons (Shelkovnikova et al., 2018). Nonetheless, it is noteworthy that in mouse embryonic stem cells, a 20-mer phosphorothioate-modified (PS) antisense oligonucleotide (ASOs) drove the assembly of PSPs into paraspeckle-like condensates lacking NEAT1 but containing the paraspeckle protein P54nrb (Shen et al., 2014). Whether such ASO-dependent paraspeckles can assemble in motor neurons needs to be established, along with evaluating the impact of such induced paraspeckles on nuclear retention and splicing functions of TDP-43. With recent regulatory approvals of several ASOs, including nusinersen (Spinraza^®^) for spinal muscular atrophy (Finkel et al., 2016; Corey, 2017), casimersen for Duchenne muscular dystrophy (DMD) (Shirley, 2021; Wagner et al., 2021) and volanesorsen for familial chylomicronemia syndrome (Gouni-Berthold et al., 2021), the prospects for therapeutic ASOs seem strong, so it will be important to exploit their potential for alleviating TDP-proteinopathies. Casimersen, is an exon-skipping ASO which functions by facilitating the expression of an internally truncated but functional dystrophin protein in DMD patients with a mutation that is amenable to exon 45 skipping (Shirley, 2021; Wagner et al., 2021). It will be interesting to determine whether similar exon skipping approaches can restore efficacious *STMN2* and *UNC13A* expression in ALS and FTLD neurons. The challenge will be specific delivery of such exon-skipping ASO to the relevant regions of the brain. Alternatively, a PS-modified version of the 34-nt TDP-43-binding oligonucleotide (bait oligonucleotide) shown by Mann et al. (2019) to enhance nuclear retention of TDP-43 may also have translational potential once targeted safe delivery to specific anatomical locations of the brain is achieved. Conceivably, computational modeling of wild type and mutant TDP-43, for instance using AlphaFold or RosettaFold algorithms (Baek et al., 2021; Jumper et al., 2021) will enable the selection of PS-rich oligonucleotides that can target TDP-43 mutants for functional retention in the nucleus. There are at least 52 missense mutations associated with ALS (Ratti and Buratti, 2016), so the ability to rapidly determine the effects of each mutation on the predicted structure of TDP-43 will provide new insights to improve the bait oligonucleotide design. A 12-nucleotide GU-repeat RNA sequence recently shown to associate with TDP-43 and prevent aggregation and localization to the cytoplasm may also hold translational potential (Rengifo-Gonzalez et al., 2021; Cascella et al., 2022).

It will also be important to determine whether ASOs or bait oligonucleotides ASOs can support the generation of the neuroprotective TDP-43 nuclear bodies that Wang et al. (2020) observed in mammalian cells and fly neurons; see NEAT1:TDP43 as protective partners in the neuronal stress response section above. Given, as mentioned above, the ability of a 20-mer PS-ASO to nucleate the assembly of PSPs into nuclear condensates lacking NEAT1 (Shen et al., 2014), can strategies be designed (for instance *via* machine learning) to uncover similar PS-ASOs that condense NEAT1 and TDP-43? In this regard, it is noteworthy that an attempt to deploy AlphaFold and other computational approaches in expanding the targetable chemical space of TDP-43 has already been reported (Scott et al., 2022). However, the recent early termination of promising ASO clinical trials for HD, due to lack of efficacy (Kwon, 2021), is a reminder of how challenging the effective deployment of ASO in any neurodegenerative disorders is likely to remain for some time.

One obvious limitation of the above approaches, however, is that while they may restore expression of functional *STMN2* or *UNC13A* they do nothing to address any pathology that may be due to cytoplasmic TDP-43 aggregates. Hence such strategies will need to demonstrate that therapeutic restoration of STMN2 or UNC13A protein expression enhances neuronal survival even in the presence of cytoplasmic TDP-43 inclusions. More pragmatically, small molecules that have shown potential as disruptors of TDP-43 aggregates, such as an acridine derivative generated by Prasad et al. (2016) are likely to be required for co-administration with ASO in order to maximize improvements in patient outcomes.

## Therapeutic horizons: Inspiration from COVID-19 messenger ribonucleic acid vaccines

The neuroprotective abilities of NEAT1_1 in TDP-43 proteinopathies reported by Matsukawa et al. (2021) aligns with earlier work showing overexpression of NEAT1_1 protected mouse neuroblasts from oxidative damage (Sunwoo et al., 2017). Upregulation of NEAT1_1 in human HD post-mortem brains was thus considered to be a neuroprotective response to limit neuronal death (Sunwoo et al., 2017). The corollary to these observations is that direct delivery of synthetic NEAT1_1 lncRNA or expression vector may help avert neuronal damage and dysfunction. The global pandemic trigger by severe acute respiratory syndrome coronavirus 2 (SARS-CoV-2) has witnessed the unprecedented deployment of mRNA in the form of mRNA vaccines, as reviewed recently (Chaudhary et al., 2021). Therefore, it should be possible to exploit similar RNA technologies in the development of NEAT1_1 for targeted delivery to neurons associated with TDP-43 proteinopathies and Huntington’s disease. The challenge will center on the development of safe and effective delivery vehicles but promising approaches for brain-targeted nucleic acid therapies (NATs) have started to emerge. These include lipid nanoparticles (Tanaka et al., 2018), cationic liposomes (Dhaliwal et al., 2020), both of which were used to deliver mRNA, and focused ultrasound (Mathew et al., 2021), which was used to deliver an expression plasmid. Clearly, these technologies can also be developed for the delivery of TDP-43 binding oligonucleotides to neurons, and it will be interesting to see which delivery system best supports the formation of neuroprotective TDP-43 nuclear bodies.

## Future horizons: Can tiny machines target transactivation response element DNA-binding protein 43 proteinopathies?

Taking inspiration from the motility of single-cell biological entities such as sperm and *Escherichia coli*, a whole new field of “swimming microrobots,” “microswimmers,” or “miniature medical robots” has emerged in recent years for a wide range of biomedical applications (Peyer et al., 2013; King, 2022). The premise of these approaches is that such untethered micromachines can deliver therapeutic payloads in a precise manner to regions of the body that are normally difficult to access and also perform minimally invasive surgical procedures (Li J. et al., 2017). Magnetic fields are arguably the most widely tested actuation methods to drive the motion of swimming microrobots (Yang and Zhang, 2020; Shao et al., 2021). However, other approaches such as ultrasound or light have also been explored, along with self-navigating methods based on chemotaxis, phototaxis, magnetotaxis, gravitaxis, and rheotaxis (Yu et al., 2021). Interestingly, swimming microrobots have become an area of intense study for targeted drug delivery to neurons (Dong et al., 2020; Kim et al., 2020). We are therefore likely to see application of these micromachines to neurodegenerative disorders in the coming years.

## Conclusion

Much progress has been made in deciphering how TDP-43 mislocalization and dysfunction contributes to neurodegeneration. As argued above, it seems that the induction of paraspeckles in the neuronal nucleus or the retention of TDP-43 as nuclear bodies can support neural viability. However, further studies are needed to clarify the relationships between induced paraspeckles and TDP-43 localization and function, especially in relation to mRNA splicing.

The emergence of NATs spanning ASO and other modalities such as short-interfering RNA (Kulkarni et al., 2021) offer a tantalizing prospect for NAT-based strategies based on oligonucleotides delivered to the brain in order to bind TDP-43 and enhance nuclear retention and restore nuclear function of TDP-43 if the promising findings from Mann et al. (2019) are reproduced in large animal models of TDP-proteinopathy. Therefore, approaches that combine TDP-43 binding oligonucleotides with studies of swimming microrobots on non-human primate models of TDP-43-mediated neurodegeneration are likely to have a transformative impact on our understanding of how to modulate the neuronal nucleus to ameliorate TDP proteinopathies.

## Author contributions

KR, DT, and DS conceptualized the idea. KR wrote the first draft. All authors read the manuscript and approved the submitted version.

## Abbreviations

AD: Alzheimer’s disease
ALS: amyotrophic lateral sclerosis
ASO: antisense oligonucleotide
CB: Cajal body
DMD: Duchenne muscular dystrophy
FTLD: frontotemporal lobar degeneration
FUS: fused in sarcoma
HD: Huntington’s disease
NEAT: nuclear enriched abundant transcript 1
NONO: non-POU domain-containing octamer-binding protein
PD: Parkinson’s disease
PSPs: paraspeckle proteins
SFPQ: splicing factor proline and glutamine rich
snRNPs: small nuclear ribonucleoproteins
STMN2: stathmin 2
TDP-43: transactivation response element (TAR) binding protein.
