# Corrigendum: TDP-43 and NEAT long non-coding RNA: Roles in neurodegenerative disease

**DOI:** 10.3389/fncel.2022.1108593

**Published:** 2022-12-16

**Authors:** Durairaj Sekar, Deusdedit Tusubira, Kehinde Ross

**Affiliations:** ^1^Centre for Cellular and Molecular Research, Saveetha Dental College and Hospitals, Saveetha Institute of Medical and Technical Sciences (SIMATS), Saveetha University, Chennai, India; ^2^Department of Biochemistry, Mbarara University of Science and Technology, Mbarara, Uganda; ^3^School of Pharmacy and Biomolecular Sciences, Liverpool John Moores University, Liverpool, United Kingdom; ^4^Institute for Health Research, Liverpool John Moores University, Liverpool, United Kingdom

**Keywords:** TDP-43, long non-coding RNA, NEAT1, neurons, paraspeckles, TAR DNA-binding protein 43, nucleic acid therapies, swimming microrobots

In the published article, there was an error. Messenger RNA was spelt out as “messenger ribosomal nucleic acid” instead of “messenger ribonucleic acid.” A correction has been made to the **Abstract**. The corrected abstract is below.

“Understanding and ameliorating neurodegenerative diseases represents a key challenge for supporting the health span of the aging population. Diverse protein aggregates have been implicated in such neurodegenerative disorders, including amyloid-β, α-synuclein, tau, fused in sarcoma (FUS), and transactivation response element (TAR) DNA-binding protein 43 (TDP-43). Recent years have seen significant growth in our mechanistic knowledge of relationships between these proteins and some of the membrane-less nuclear structures that fulfill key roles in the cell function. These include the nucleolus, nuclear speckles, and paraspeckles. The ability of macromolecular protein:RNA complexes to partition these nuclear condensates through biophysical processes that involve liquid–liquid phase separation (LLPS) has also gained attention recently. The paraspeckle, which is scaffolded by the architectural long-non-coding RNA nuclear enriched abundant transcript 1 (NEAT1) plays central roles in RNA processing and metabolism and has been linked dynamically to TDP-43. In this mini-review, we outline essential early and recent insights in relation to TDP-43 proteinopathies. We then appraise the relationships between TDP-43 and NEAT1 in the context of neuronal paraspeckles and neuronal stress. We highlight key areas for investigation based on recent advances in our understanding of how TDP-43 affects neuronal function, especially in relation to messenger ribonucleic acid (mRNA) splicing. Finally, we offer perspectives that should be considered for translational pipelines in order to improve health outcomes for the management of neurodegenerative diseases.”

In the published article, there was an error. Messenger RNA was spelt out as “messenger ribosomal nucleic acid” instead of “messenger ribonucleic acid.” A correction has been made to the **Introduction**, paragraph 4. The corrected paragraph is below.

“Transactivation response element (TAR) DNA-binding protein 43 is a highly conserved heterogeneous ribonucleoprotein (hnRNP) multi-domain protein first identified as a 43-kDa protein that bound the TAR in human immunodeficiency virus (Ou et al., 1995). Under normal physiological conditions, TDP-43 is subjected to nucleocytoplasmic shuttling while residing predominantly in the nucleus (Ayala et al., 2008). This localization to both nuclear and cytosolic compartments is reflected in the processes regulated by TDP-43, which span messenger ribonucleic acid (mRNA) transcription splicing, maturation, and mRNA transport as well as the formation of stress granules and the regulation of miRNA processing, as reviewed recently by Prasad et al. (2019). Unsurprisingly, therefore, mutations that increase TDP-43 aggregation, increase TDP-43 half-life, or alter TDP-43 interactions with other proteins are thought to contribute to disease pathology in TDP-43 proteinopathies, and over 52 TDP-43 mutations have been linked to disease (Buratti, 2015).”

In the published article, there was an error. Messenger RNA was spelt out as “messenger ribosomal nucleic acid” instead of “messenger ribonucleic acid.” A correction has been made to the section heading “**Therapeutic horizons: Inspiration from COVID-19 messenger ribosomal nucleic acid vaccines**.” The corrected heading is “**Therapeutic horizons: Inspiration from COVID-19 messenger ribonucleic acid vaccines**.”

The authors apologize for this error and state that this does not change the scientific conclusions of the article in any way. The original article has been updated.
